# Identification of differentially accumulated proteins associated with embryogenic and non-embryogenic calli in saffron (*Crocus sativus *L.)

**DOI:** 10.1186/1477-5956-10-3

**Published:** 2012-01-13

**Authors:** Golandam Sharifi, Hassan Ebrahimzadeh, Behzad Ghareyazie, Javad Gharechahi, Elaheh Vatankhah

**Affiliations:** 1 Department of Basic Sciences, Iranian Encyclopedia Compiling Foundation, Tehran, Iran; 2 Department of Botany, Faculty of Science, University of Tehran, Tehran, Iran; 3 Department of Genomics, Agricultural Biotechnology Research Institute of Iran, Karaj, Iran; 4 Department of Molecular Genetics, National Institute for Genetic Engineering and Biotechnology, Tehran, Iran

**Keywords:** Saffron, *Crocus sativus *L., somatic embryogenesis, two-dimensional gel electrophoresis, MALDI-TOF/TOF

## Abstract

**Background:**

Somatic embryogenesis (SE) is a complex biological process that occurs under inductive conditions and causes fully differentiated cells to be reprogrammed to an embryo like state. In order to get a better insight about molecular basis of the SE in *Crocus sativus *L. and to characterize differentially accumulated proteins during the process, a proteomic study based on two-dimensional gel electrophoresis and matrix-assisted laser desorption/ionization time of flight mass spectrometry has been carried out.

**Results:**

We have compared proteome profiles of non-embryogenic and embryogenic calli with native corm explants. Total soluble proteins were phenol-extracted and loaded on 18 cm IPG strips for the first dimension and 11.5% sodium dodecyl sulfate-polyacrylamide gels for the second dimension. Fifty spots with more than 1.5-fold change in abundance were subjected to mass spectrometry analysis for further characterization. Among them 36 proteins could be identified, which are classified into defense and stress response, protein synthesis and processing, carbohydrate and energy metabolism, secondary metabolism, and nitrogen metabolism.

**Conclusion:**

Our results showed that diverse cellular and molecular processes were affected during somatic to embryogenic transition. Differential proteomic analysis suggests a key role for ascorbate metabolism during early stage of SE, and points to the possible role of ascorbate-glutathione cycle in establishing somatic embryos.

## Background

Saffron (*Crocus sativus *L., Iridaceae) has long been cultivated for the production of saffron spice, which makes it interesting from an economic as well as a scientific point of view. Saffron is an autumn flowering species and an auto-triploid (2n = 3x = 24) form of a species found in eastern Greece. An origin in Western or central Asia (possibly Iran) is suspected [1]. *In vitro *culture of saffron is a promising approach for making the commercial production of crocin, safranal and picrocrocin (the flavor and coloring characteristic of saffron) less expensive than conventional means i.e. through manual harvesting of styles [2]. Somatic embryogenesis (SE) has also been recognized as a promising approach for the regeneration of plantlets in tissue cultures and as a vegetative propagation system *in vitro*. The ability of plant cells to produce somatic embryos in culture, made SE not only as an interesting issue for genetic engineering and biotechnology but also as a model system for studying zygotic embryogenesis [3,4]. Several potential biotechnological applications e.g. artificial seeds, micropropagation, germplasm conservation, transgenic plants, etc. have been reported for SE [4]. Somatic embryos have been demonstrated to be morphologically and developmentally similar to their zygotic counterparts and they both proceed through a series of distinct stages, i.e. globular, heart, torpedo, and cotyledon stages for dicotyledons and globular, elongated, scutelar, and coleoptilar stages for monocotyledons [4-6].

Plant growth regulators (PGRs) have a critical role in SE induction and subsequent modulation of the proper morphogenesis in embryo development. Depending on the nature of the explant, auxin and/or cytokinin may be used to induce SE in culture [7-9]. However, decreasing or removal of exogenous auxin is necessary for embryo morphogenesis and further development [5]. During SE, differentiated somatic cells undergo a series of morphological and biochemical changes and are completely reprogrammed to an embryonic like state which forms the basis of cellular totipotency in plants [8]. Transition from an unstructured callus to the somatic embryo, a highly organized structure, requires global changes in the gene expression to support this developmental switching. Thus, understanding the molecular and biochemical pathways that initiate and direct vegetative to embryogenic transition is of great importance to plant molecular biologists.

Quantitative and qualitative analysis of transcriptomic and proteomic changes associated with SE could be considered as an important step towards the elucidation of underlying mechanism(s) of SE. High throughput analyses of gene expression at the mRNA level have provided a wealth of information about the genes that are involved in SE in different plant species [10-16]. Several gene classes associated with SE including auxin-related genes [17-19], ABA-inducible genes [20], SERK genes [9,20], calmodulin [21], LEC genes [22,23], AP2/ERF family [24,25], WUSCHEL [26] and AGL15 [27] have been identified. Although mRNA expression profiling has been proven as a powerful tool, this approach suffers from some inherent limitations. There is no clear correlation between mRNA and protein abundance, due to the variation in mRNA stability, translatability, and protein stability [28,29]. Furthermore, protein structure, activity, and function can be altered and regulated by subcellular localization, interaction by other molecules, and posttranslational modifications that would not be detected by mRNA analysis [30]. Consequently, there is a growing recognition that whilst mRNA expression profiling continues to be a valuable tool, this approach should be complemented with profiling methods of the final gene products or proteins themselves.

Proteomics has been defined as the systematic analysis of proteins expressed by a genome at a definite point in time [31]. Proteomics is a powerful approach to study plant responses to various biotic and abiotic stresses, and biochemical changes associated with developmental pathways [32]. A comprehensive protein expression profile can be analyzed and compared using a 2-DE based protein separation method combined to mass spectrometry based protein identification system. There are several proteomics reports dealing with SE in different plant species e.g. *Daucus carota *[33], *Oryza sativa *L. [34], *Camellia japonica *[35], *Cupressus sempervirens *L. [36], *Spinacia oleracea *[37], *Vitis vinifera *[38], *Medicago truncatula *[7,39], *Cyclamen persicum *[40-42], *Picea glauca *[43], *Citrus sinensis *Osbeck [44,45], and *Acca sellowiana *[46]. Proteomic analyses provide new insights into the molecular basis of SE and exploring some black boxes of this process, pave the way for future *in vitro *scale up propagation and genetic manipulation through the development and optimization of strategies for efficient somatic embryo production.

To date there has been no report on systematic analysis of transcriptome and proteome in saffron. To the best of our knowledge, this is the first report that uses two-dimensional gel electrophoresis in combination with tandem mass spectrometry to evaluate the proteomic changes that occur during SE induction in saffron. We aimed to identify proteins that are differentially modulated during SE induction in saffron. Mass spectrometry analysis led to the identification of 36 differentially accumulated proteins. The possible implications of the differentially accumulated proteins in SE induction were discussed.

## Materials and methods

### Plant materials and tissue culture

Qaen saffron (the accession that had been collected from farms of the Qaen city in south Khorasan province, Iran) was used as the starting plant material. Healthy resting corms, which were growing in the research farm of the University of Tehran (Mardabad, Karaj, Iran), were collected in August, and washed under running tap water for 30 min. After surface disinfection with detergent, they were soaked in hygiene (1% benzalkonium chloride) for 10 min, and then were rinsed under tap water. The corm explants were transferred to a sterile laminar airflow cabinet, incubated first in 70% ethanol for 2 min then in 20% (v/v) commercial bleach containing 1% sodium hypochlorite for 15 min and rinsed three times in distilled sterile water. The basal medium used for tissue culture was Murashige and Skoog [47]. The culture medium was supplemented with 30 g/l sucrose and solidified with 7 g/l agar (BactoAgar, Difco Laboratories). The pH was adjusted to 5.7 with 1 M NaOH prior to autoclaving. The culture medium was autoclaved at 120°C for 20 min. After cooling the media (50°C), plant growth regulators that had been dissolved in DMSO were added and media were distributed in culture dishes.

A rectangular section from the central meristematic region of the corms was used as the starting explant. Twenty five explants (five in each plate) were placed on solidified culture medium supplemented with 1 mg/l 2,4-D and 4 mg/l Kin. The dishes were incubated at 25 ± 3°C temperature regime in the dark. At the same time, some explants from different corms were pooled in three replicates frozen in liquid nitrogen and stored at -80°C for further analysis. After 5 to 6 weeks in this culture condition, they started developing embryogenic calli (nodular calli, NC). Nodular calli were calli that contained globular stage embryos. After four subcultures (four weeks interval), the cultures were analyzed and all calli were screened visually based on their morphology. During these time intervals, some calli remained amorphous and did not develop any embryo like structures (non-embryogenic calli, NEC). The percentage of total calli and nodular calli induction frequencies were calculated based on Pearson χ^2 ^test. Both embryogenic (NC) and non-embryogenic calli (NEC) were harvested in three replicates frozen in liquid nitrogen, and stored at -80°C until use.

### Protein extraction

Protein extraction was performed as described by Hurkman and Tanaka [48] with some modifications. Briefly, plant material was ground in liquid nitrogen using mortar and pestle. The resulting powder was transferred to a 10 ml tube. Then 2.5 ml extraction buffer (0.1 M Tris-HCl, pH 8.8; 10 mM EDTA; 0.4% 2-mercaptoethanol and 0.9 M sucrose) was added to each tube, after brief vortexing, 2.5 ml Tris pH 8.8 buffered phenol (Sigma, St. Louis, MO, USA) was added. After vortexing for 30 min at 4°C, centrifugation was carried out in 5000 × g at 4°C for 10 min. The upper phenol phase was carefully decanted and transferred to a new clean tube. These steps were repeated for the remaining aqueous phase by adding 2.5 ml Tris buffered phenol. Proteins in the collected phenol phase were precipitated by adding five volumes of pre-chilled 0.1 M ammonium acetate in 100% methanol and incubation at -20°C. The precipitate was collected by centrifugation for 20 min, 20000 × g at 4°C. Finally, the pellet was washed 2 times with 0.1 M ammonium acetate in methanol, 2 times with ice-cold 80% acetone and finally 1 time with cold 70% ethanol. After a brief air-drying, the protein pellet was re-suspended in lysis buffer (8 M Urea, 2 M Thiourea, 4% CHAPS, 50 mM DTT, 35 mM Tris and 2% pharmalyte (pH 3-10). Total protein concentration was quantified by Bradford assay [49] using IgG as the standard.

### Two-dimensional gel electrophoresis (2-DE)

Total protein extract (160 μg) was loaded onto 18 cm IPG gel strips (pH 4-7, Bio-Rad, Hercules, CA, USA) during strip rehydration overnight. IEF was then performed for a total of 52 kVh at 20°C using Multiphore ΙΙ system (Amersham Pharmacia Biotech, Uppsala, Sweden). The IPG strips were equilibrated according to the manufacturer's instruction in a solution containing (50 mM Tris-HCl buffer, pH 8.8, 6 M w/v urea, 30% v/v glycerol, 2% w/v SDS, 1% w/v DTT, 0.002% of bromophenol blue). The second dimension was performed on 11.5% SDS-polyacrylamide gel using a Protean Dodeca Cell (Bio-Rad, Hercules, CA, USA) at 50 V for 30 min and then at 200 V for about 7 h at 4°C. In analytical phase, gels were stained using silver nitrate according to Blum et al. [50], and in preparative phase gels were stained by coomassie brilliant blue (CBB) G250 [51]. Each treatment was run in three biological replicates.

### Image acquisition and data analysis

Gel images were acquired using a GS800 calibrated densitometer (Bio-Rad, Hercules, CA, USA) at a resolution of 700 dpi. The scanned gels were saved as TIFF images for subsequent analysis. Image treatment, spot detection, and quantification were carried out using Melanie 6.02 software (GeneBio, Geneva, Switzerland). The spot detection parameters were set by checking different protein spots in certain regions of the gels, followed by visual inspection for deletion or addition of spot artifacts and undetected spots, respectively. The processed gels were automatically matched to attribute a common spot identity for the same spot derived from different gels and visually inspected for improper spot matches. The volume of each spot from three replicate gels was normalized against total spot volume, and the resulting percent volumes (%Vol) were subjected to Student's t-test (*p *≤ 0.05) for statistical analysis.

### Spot excision and in-gel trypsin digestion

The spots displaying more than 1.5-fold change in abundance were selected for further characterization using MS. Spots were manually excised from preparative CBB stained gels and were analyzed using MALDI-TOF/TOF mass spectrometry at the Proteomics Laboratory, University of York, UK. Protein spots were washed three times with ultrapure water and then destained twice with 50% (v:v) aqueous acetonitrile containing 25 mM ammonium bicarbonate, followed by one wash with acetonitrile. After washing, gel pieces were left to dry in a vacuum concentrator for 20 min. Sequencing-grade, modified porcine trypsin (Promega) was dissolved in 50 mM acetic acid supplied by the manufacturer, then diluted 5-folds by adding 25 mM ammonium bicarbonate to a final trypsin concentration of 0.01 μg/μl. Gel pieces were rehydrated by adding 10 μl of trypsin solution, and after 30 min, enough 25 mM ammonium bicarbonate solution was added to cover the gel pieces. Digestion reaction was incubated overnight at 37°C.

### MALDI-TOF/TOF MS analysis and database searching

One μl aliquot of each peptide mixture was applied directly to the ground steel MALDI target plate, then an equal volume of a freshly-prepared 5 mg/ml solution of 4-hydroxy-α-cyano-cinnamic acid (Sigma) in 50% aqueous (v:v) acetonitrile containing 0.1%, trifluoroacetic acid (v:v) was added. Positive-ion MALDI mass spectra were obtained using a Bruker ultraflex III in reflectron mode, equipped with a Nd:YAG smart beam laser. MS spectra were acquired over a mass range of m/z 800-4000. Final mass spectra were externally calibrated against an adjacent spot containing six peptides (des-Arg^1^-Bradykinin, 904.681; Angiotensin I, 1296.685; Glu^1^-Fibrinopeptide B, 1750.677; ACTH (1-17 clip), 2093.086; ACTH (18-39 clip), 2465.198; ACTH (7-38 clip), 3657.929.). Monoisotopic masses were obtained using a SNAP averaging algorithm (C 4.9384, N 1.3577, O 1.4773, S 0.0417, H 7.7583) and a S/N threshold of 2. Ten of the strongest peaks of interest, with an S/N greater than 30, were selected for MS/MS fragmentation for each spot. Fragmentation was performed in LIFT mode without the introduction of a collision gas. The default calibration was used for MS/MS spectra, which were baseline-subtracted and smoothed (Savitsky- Golay, width 0.15 m/z, cycles 4); monoisotopic peak detection used a SNAP averagine algorithm (C 4.9384, N 1.3577, O 1.4773, S 0.0417, H 7.7583) with a minimum S/N of 6. Bruker flex Analysis software was used to perform the spectral processing and peak list generation for both the MS and MS/MS spectra. The mass spectral and tandem mass spectral data were submitted to database searching using a locally-running copy of the MASCOT program (Matrix Science Ltd., version 2.1), through the Bruker BioTools interface (version 3.2). Search criteria were as follows: database, NCBInr; taxonomy, Viridiplantae (green plants); enzyme, trypsin; fixed modifications, carbamidomethyl (C); variable modifications, oxidation (M); peptide tolerance, 100 ppm; MS/MS tolerance, 0.5 Da; instrument, MALDI-TOF/TOF (NCBInr 20090906 (9655479 sequences; 3300246437 residues)). The threshold for positive identification was a MOWSE score of > 71(*p *≤ 0.05).

### Statistical analysis

Differences in the percentages of callus formation were statistically compared by cross tabulation and calculation of Pearson χ^2 ^using SPSS software version 14.0 (SPSS, Chicago, IL, USA). A two-tailed Student's t-test in Excel medium (Microsoft Office Excel) was employed to compare relative protein abundance in proteomic analysis.

## Results and discussion

### Tissue culture and somatic embryogenesis

**Figure 1:**
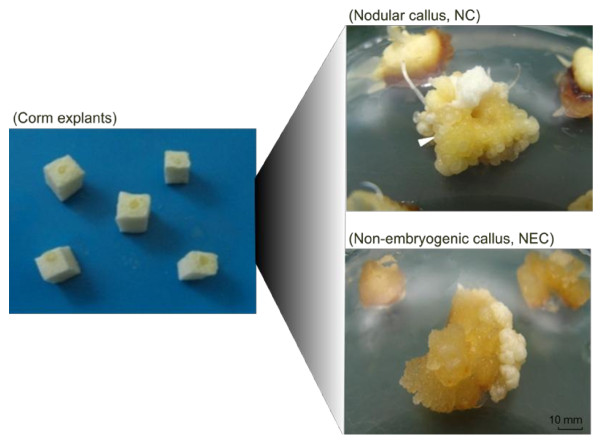
**Somatic embryogenesis in saffron**. Rectangular sections of the meristematic tissue of corm collected in August were used as the initial explants. SE was induced in MS medium containing auxin (2,4-D) and cytokinin (Kin). Corm explants produced both embryogenic (Nodular callus, NC) and non-embryogenic (NEC) calli. NC were nodular and translucent in color, while NEC were amorphous and spongy. The characteristic feature of the globular embryos was that they easily dissociated from mother calli upon touch. White arrow shows the location of a globular stage embryo.

Since saffron is a sterile plant (triploid), clonal propagation through SE is considered as an alternative approach to the conventional harvesting of styles for the commercial production of saffron metabolites, which have broad pharmaceutical and coloring properties. Hence, developing efficient protocols for saffron SE would open new avenues to the pharmaceutical industry. The first report of saffron tissue culture dates back to work carried out by Ding and colleagues [52]. They used corm as the early explant and successfully regenerated intact plantlets in a culture media supplemented with IAA and 2,4-D as PGRs. Here SE was induced from meristematic section of the corm explants cultured on Murashige and Skoog medium containing 2,4-D and kinetin. To assess callugenesis, total and nodular callus induction frequencies were calculated which were 62 and 18%, respectively, after 16 weeks in culture. NC appeared nodular and translucent in color with smooth surface and no hair like structures, while non-embryogenic calli (NEC) were spongy and amorphous. The process of tissue culture and morphology of NC and NEC are shown in Figure 1.

### Protein extraction and 2-DE analysis

**Figure 2:**
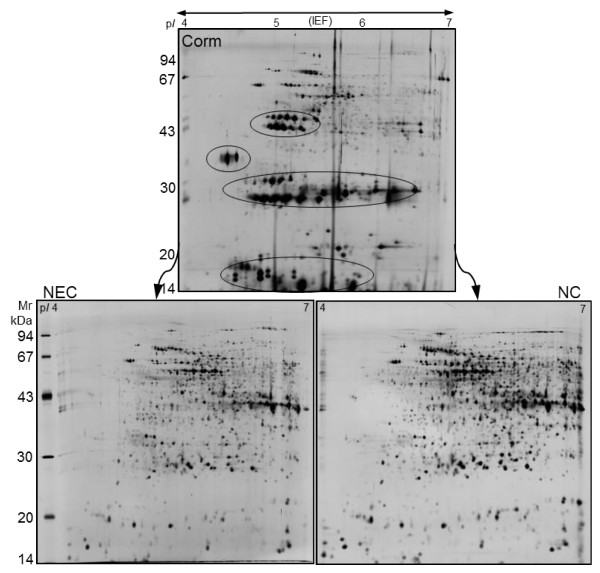
**Comparison of 2-DE gel images derived from corm explants along with its embryogenic (NC) and non-embryogenic (NEC) calli after SE induction in culture**. The black circles show the location of the highly abundant protein spots that completely vanished or decreased in abundance during somatic to embryonic transition.

To study proteins modulated during somatic to embryogenic transition, corm-derived explants along with non-embryogenic calli (NEC), which did not have the embryo like structures, and nodular calli (NC), which contained globular stage embryos, were used for protein extraction and proteomic analysis (Figure 1). Protein extraction and solubilization are critical steps for successful gel-based proteomic analysis. Due to the high phenolic content of callus material protein extraction presents a major challenge. We tried two different protein extraction methods (TCA acetone precipitation [53], and phenol extraction [48]) and found that in agreement with previous results [54] phenol extraction method gives highly resolved gels with more detectable spots. Total soluble proteins were extracted from corm explants, NEC and NC and were resolved by 2-DE. Figure 2 displays representative gel images of 2-DE proteome pattern of corm explants, NEC and NC.

**Figure 3:**
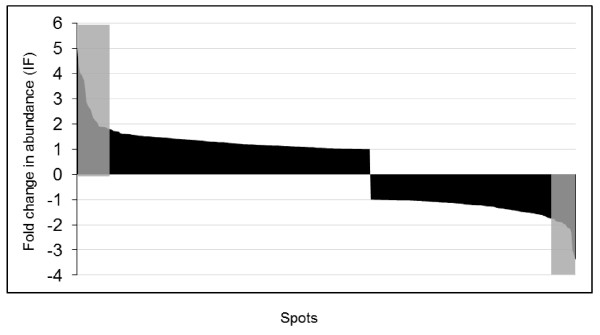
**Graph shows the frequency distribution of the relative abundance ratios (induction factors) for all matched spots between NC and NEC gels**. The highlighted regions show the statistically significant spots that showed more than 1.5 fold change (increase or decrease) in abundance in NC compared with NEC.

**Figure 4:**
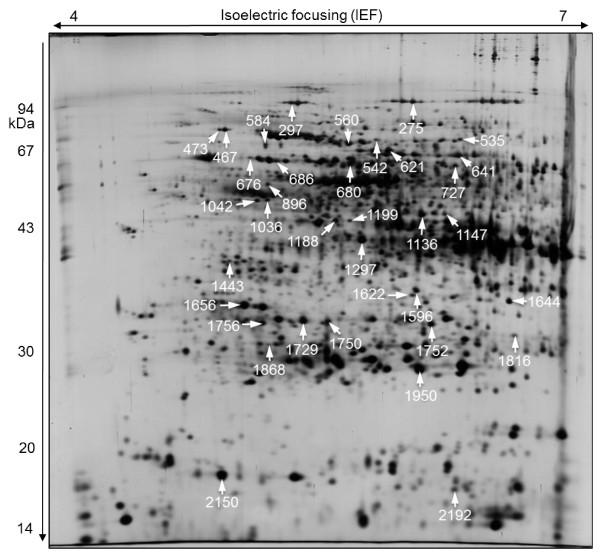
**2-DE map of proteins extracted from NEC after SE induction *in vitro***. Total soluble proteins were extracted by phenol extraction method; 160-μg protein was loaded into 18 cm IPG strips with linear pH gradient of 4-7 for isoelectric focusing (IEF). Second dimension was run in 11.5% SDS-PAGE gels. Proteins were visualized by silver staining. Arrows show the positions of the identified proteins by MS, which showed statistically significant change in abundance. The numbering corresponds to the match IDs as shown in table 1.

Image analysis revealed that the proteome profile of the corm explant was significantly different compared to its derived NEC and NC (Figure 2). Roughly, 600, 850, and 800 reproducible spots could be detected in the corm, NEC, and NC 2D gels, respectively. There were many high abundant protein spots in the corm explants gels in the range of 14-20, 30, and 43 kDa that were either entirely absent or had low abundance in its resultant NC and NEC (Figure 2). When proteomes of NC and NEC were compared the majority of the protein spots had similar abundance and only 9 percent showed differences, indicating that the majority of the proteins were not changed in NC compared with NEC calli. Sixty-five spots were statistically significant (*p *≤ 0.05) and showed more than 1.5-fold change in abundance (Figure 3). As it is shown in the graph (Figure 3), spots with increasing trend in abundance are dominant. Among the identified proteins, twenty appeared to be increased or decreased in abundance in NEC and NC in relation to the original corm explants. Sixteen protein spots (676, 1729, 1147, 1443, 1868, 1644, 584, 621, 1622, 1656, 1750, 1752, 1950, 2150, 2192, and 1596) appeared to be absent in the corm explants derived gels. More than 94% of the identified proteins showed an increase in abundance in NC and NEC compared with their original corm explants. Table 1 shows the list of the identified proteins with their respective spot ID, theoretical and experimental isoelectric point (p*I*) and molecular weight (MW), protein identity and accession number, MS score, percent of sequence coverage, PMF/MS-MS and abundance ratio. The position of the identified protein spots are shown in gel image Figure 4. In most cases, the theoretical MWs agreed well with experimental values except for spot 1868 that had lower experimental MW, which may be due to the possible protein degradation. Clear correlation was not seen between theoretical and experimental p*I*s, because experimental p*I*s were directly estimated from gel images that are subjected to perturbation due to inconsistency in pH gradient across gel strips and variation in the protein migration during the first dimension. Interestingly, for spots 1729, 584, 1656, 1752, and 1756 theoretical p*I*s were largely deviated from that of corresponding experimental ones. The observed deviations may also be due to either the possible posttranslational modifications or the fact that the identified proteins belonged to the species other than *C. sativus*. The determination of p*I *directly from protein migration in gels has been found less accurate than MW [43,46].

**Table 1:** Differentially modulated proteins (≥ 1.5-fold change in abundance) in embryogenic (NC) and non-embryogenic (NEC) calli compared with corm explants in *Crocus sativus*.

Spot ID^a^	The/Exp^b^		Protein name/Organism	Accession No^c^	% Cov./Scor.^d^	PMF/MS-MS^e^	Abundance ratio^f^		
	**MW** **(kDa)**	**p*I***					**NC/** **Corm**	**NEC/** **Corm**	**NC/** **NEC**
						
467	75/80	5/4.89	Heat shock protein 70/*Cucumis sativus*	Q39641	81/556	9/5	1.9**	1.6*	1.1
473	75/79	5/4.85	Heat shock protein 70/*Cucumis sativus*	Q39641	12/402	6/5	1.6*	1.6*	1.0
535	61/73	5.7/6.27	Heat shock protein 70 (HSP70)-interacting protein, putative/*Ricinus communis*	B9RBP6	8/98	4/1	5.6**	3.6*	1.5*
542	62/73	5/5.76	Heat shock 70 kDa protein, mitochondrial/*Solanum tuberosum*	Q08276	18/88	10/1	5.8**	3.6**	1.6**
560	72/70	5.5/5.59	Heat shock 70 kDa protein/*Zea mays*	B6U4A3	19/375	12/4	6.3**	5.1**	1.2
680	59/61	5.4/5.61	T-complex protein 1 epsilon subunit, putative/TCP-1-epsilon/*Arabidopsis thaliana*	O04450	8/101	4/3	5.4**	7.4**	-1.4
686	63/62	5.5/5.20	Chaperonin/*Arabidopsis thaliana*	Q9LJE4	17/226	7/3	-1.5	-1.6*	1.1
727	57/61	5.5/6.24	Chaperonin/*Arabidopsis thaliana*	Q940P8	20/272	7/4	6.9**	5.3*	1.3
641	60/64	5.97/6.27	Chaperonin containing t-complex/*Ricinus communis*	B9SUJ3	18/137	9/3	6.1**	5.1*	1.2
676	62/63	6.6/5.03	Rubisco large subunit-binding protein/*Brassica napus*	P21241	10/96	4/2	˃	˃	1.3
1188	47/50	6.9/5.55	Glutamine synthetase precursor/*Glycine max*	Q95AG1	16/378	7/4	5.6**	4.6**	1.2
1297	39/47	5.3/5.73	Glutamine synthetase/*Raphanus sativus*	O24334	17/310	5/3	4.4**	3.8**	1.2
1729	20/34	9.7/5.37	Glutathione S-transferase/*Gossypium barbadense*	A7KP03	6/79	1/1	˃	˃	-1.4*
1656	20/36	9.7/5.01	Glutathione S-transferase/*Gossypium barbadense*	A7KP03	6/86	1/1	˃	˃	-1.2
1950	26/29	6.2/6.04	Glutathione S-transferase U20/*Arabidopsis thaliana*	Q8L7C9	11/80	3/2	˃	˃	-1.3
1036	47/54	4.8/5.15	26S protease regulatory subunit 6A homolog/*Solanum Lycopersicum*	P54776	27/541	15/7	4.0**	3.8*	1.0
1042	47/54	4.8/5.08	26S protease regulatory subunit 6A homolog/*Solanum Lycopersicum*	P54776	29/516	16/9	5.9**	4.3**	1.4
1136	42/51	6/6.05	dTDP-glucose 4-6-dehydratase/*Ricinus communis*	B9SZ78	35/381	15/4	3.0*	4.2**	-1.4
1147	42/51	5.8/6.18	GDP-D-mannose-3',5'-epimerase/*Malpighia glabra*	A0EJL8	15/108	6/3	˃	˃	1.4*
275	99/99	5.8/6.03	Aconitase/*Ricinus communis*	B9SXB6	7/85	6/2	9.1**	8.6**	1.0
1756	34/33	9.7/5.12	6-phosphogluconolactonase, putative/*Ricinus communis*	B9RWU6	12/86	2/1	-9.1**	-7.7**	1.2
1443	35/43	4.9/4.91	Probable fructokinase-2/*Arabidopsis thaliana*	Q9LNE3	22/182	7/3	˃	˃	1.2
1199	51/50	5.8/5.61	Elongation factor Tu, chloroplastic/*Arabidopsis thaliana*	P17745	16/360	5/5	1.8**	1.4^ns^	1.3
1868	92/31	5.8/5.14	Initiation factor eIF-4 gamma, middle; Up-frameshift suppressor2/*Medicago truncatula*	Q2HSQ9	63/72	5/0	˃	˃	-1.4*
1644	32/37	5.69/6.56	Isoflavone reductase-like1/*Zea mays*	P52580	7/85	2/1	˃	˃	-1.1
1596	33/39	5.76/6.04	Isoflavone reductase-like protein 5/*Vitis vinifera*	Q3KN68	15/123	3/1	˃	˃	-1.1
1622	34/38	6/6.02	Isoflavone reductase related protein/*Pyrus communis*	O81355	11/147	2/2	˃	˃	1.2
896	45/57	5.9/5.14	RNA binding protein 45/*Nicotiana plumbaginifolia*	Q9LEB4	3/88	1/1	4.7*	5.2*	-1.1
297	90/97	5.28/5.13	Cell division control protein 48 homolog A/*Arabidopsis thaliana*	P54609	22/238	12/5	4.2*	3.7**	1.1
1816	22/33	5.9/6.72	Cysteine proteinase inhibitor 6/*Arabidopsis thaliana*	Q8H0X6	25/155	5/2	2.9*	3.7**	1.2
584	84/70	9.4/5.1	Putative uncharacterized protein/*Oryza sativa *Japonica Group	B9FCS8	6/121	4/3	˃	˃	1.4
621	61/67	5.29/5.7	Phosphoglyceromutase/*Mesembryanthemum crystallinum*	Q42908	13/191	5/2	˃	˃	1.2
1750	27/34	5.2/5.5	Caffeoyl-CoA O-methyltransferase/*Solanum tuberosum*	Q8H9B6	45/488	10/4	˃	˃	-2.7**
1752	42/35	9.3/6.1	Ascorbate peroxidase/*Lycopersicon esculentum*	Q8LSK6	20/268	6/3	˃	˃	1.1
2150	8.8/19	4.8/4.8	Copper chaperone/*Zea mays*	B6T1K0	32/211	3/3	˃	˃	-1.4
2192	36/18	6.3/6.2	Cys/Met metabolism PLP-dependent enzyme family protein/*Oryza sativa *(japonica cultivar-group)	Q10KP3	13/76	6/0	˃	˃	-2*

a) The numbering corresponds to the match IDs in 2D gels.

b) Theoretical/Experimental MW (kDa) and p*I*.

c) Accession number in Uni-Prot.

d) Percent of sequence coverage and Mascot score resulted from combined MS-MS/MS search.

e) Number of peptide identified by PMF and MS/MS.

f) Fold change in abundance levels, * (*p *≤ 0.05), ** (*p *≤ 0.01)

### Identification and functional classification of SE-associated proteins

Finally, differentially modulated protein spots (with 1.5 fold change in abundance) were selected and manually excised from 2D CBB-stained gels and were subjected to in-gel trypsin digestion and MALDI-TOF/TOF tandem mass spectrometry identification. Protein identification was carried out by combined PMF and MS/MS approach. Search was performed against non-redundant protein database at the NCBI. Of the 50 candidate spots analyzed by mass spectrometry only 36 proteins (72%) were successfully identified, which showed increase or decrease in abundance (Table 1). For the remaining spots a low score or no hits were observed. Owing to the lack of sequence information from *C. sativus *in the databases, all identified proteins belonged to other species mainly *Arabidopsis thaliana *and *Ricinus communis*. The percentages of sequence coverage of the identified proteins were 3-80%. Only heat shock 70 kDa interacting protein (535) and mitochondrial heat shock 70 kDa (542) increased in NC compared to NEC. Glutathione S-transferase (1729), initiation factor eIF-4 gamma (1868), caffeoyl-CoA O-methyltransferase (1750), and Cys/Met metabolism PLP-dependent enzyme (2192) were specifically decreased in NC compared to NEC.

**Figure 5:**
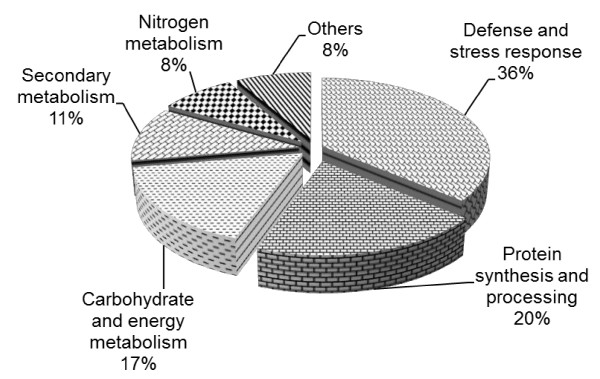
**Functional classification of the differently accumulated proteins during SE induction in *C. sativus***. The pie chart shows the distribution of the proteins which appeared to be increased or decreased during SE into different functional classes in percentage.

It is important to note that proteins with the same name might be found in more than one spot. For example, we found that, spots 467, 560 and 473 which were identified as hsp70, spots 1188 and 1297 which were identified as glutamine synthetase, and spots 1036 and 1042 which were identified as 26S protease regulatory subunit 6A homolog shifted slightly in p*I *and were seen as spot train in 2D gels (Figure 4). This indicates the presence of multiple differentially charged isoforms which are commonly observed with abundant proteins. Interestingly, spots 686 and 717 which were identified as chaperonin had the same MW but very different positions horizontally, suggesting that they may be posttranslationally modified [55]. As a consequence, 29 distinct protein species were identified. Identified proteins were classified into five functional groups based on their main biological process http://www.uniprot.org: defense and stress response (13 spots), protein synthesis and processing (7), carbohydrate and energy metabolism (6), secondary metabolism (4), and nitrogen metabolism (3) (Figure 5).

### Differentially accumulated proteins in embryogenic and non-embryogenic calli

The objective of this study was to gain insight into the molecular and biochemical changes associated with SE induction in saffron, which in turn can be useful for the development of efficient SE protocols. Although, various gene classes have been identified to be involved in SE [20] but there have only been little successes in finding early genes whose expression support SE induction [7]. Interestingly, differentially accumulated proteins of several different functional categories were observed in this study. The majority of the protein species identified correspond to enzymes involved in oxidative stress, metabolic processes, and protein synthesis and degradation, and some of them have not been previously described in the context of SE. It should be noted, however, that the levels of some of these proteins, especially defense-related proteins, might be affected by tissue culture conditions *in vitro *[56,57]. In the present study, identification of the candidate proteins was relied essentially on homology search to known sequences of the other plant species because of the poor genome and protein sequence information that is currently available for *Crocus sativus*.

SE is a complete cell reprogramming process that would be associated with complex changes in gene expression and proteome profile [5,57]. In agreement with this, we found that the proteome profiles of NC and NEC were significantly different compared to the original corm explants, which specifies complete reprogramming of gene expression taking place to support somatic to embryogenic transition. Our results indicate that the initial stage of dedifferentiation of somatic cells to embryo-like structure needs global change in gene expression and protein complement. Of the fifty candidate proteins which were analyzed by MS, we were able to identify only 36 proteins, due largely to the limitation of the databases used for MS data mining. We will discuss our proteomic results based on the functional classification of the differentially accumulated proteins as shown in Figure 5.

### Defense and stress response proteins

In this study based on gene ontology classification, 13 proteins (36%) were categorized as defense and stress response proteins. Gene expression analyses at both proteome and transcriptome levels have led to the identification and characterization of some stress-related genes and proteins associated with SE. Interestingly, some reports highlight that somatic embryogenesis itself is induced by oxidative stresses [15,57]. However, it is important to take into account that the higher abundance of some of the proteins involved in defense and stress responses might be evoked by the stresses associated with tissues wounding during explant preparation and subculture.

As stress responsive proteins, we found four heat shock proteins 70 (HSPs70) (spots 467, 473, 542 and 560), all significantly accumulated in embryogenic (NC) and non-embryogenic (NEC) calli. In case of spots 535 and 542, an increase in abundance was observed in NC compared to NEC. Heat shock proteins (HSPs) are a class of highly abundant proteins that are expressed upon elevated temperature and many other stresses. Similar to our results, HSPs proteins have been reported to be highly increased during somatic embryo maturation and germination of Cork oak [58] and SE of *Vitis vinifera *[38], somatic and zygotic embryos of *Cyclamen persicum *[41]. dnaK-type hsp70 and mitochondrial hsp70 have been found to be highly accumulated in the early stages of SE in *Medicago truncatula *[7] and *Picea glauca *[43], respectively. Taken together, the data presented in this work suggest that the increased chaperone proteins may play a key role in SE possibly by alleviating stresses associated with global reprogramming during somatic to embryogenic transition. The results are consistent with other studies showed that HSPs were differentially regulated during somatic embryo development in response to hormones such as 2,4-D [59,60]. Differential regulation of HSP genes in some circumstances may cause growth arrest in globular embryos but not somatic embryos at other developmental stages [61-63]. Although heat shock proteins are considered as stress responsive proteins, however, many of them are expressed during normal cell growth and function as chaperone aiding in protein folding and subcellular sorting.

Among the candidate proteins, three (spots 1729, 1950, and 1656) were identified as glutathione S-transferase (GST). GST appeared to be absent in the corm-derived gels. It showed a slight decrease in NC compared with NEC, suggesting that this enzyme is an early responsive protein to SE. Consistently, GST accumulation has been reported in somatic embryos of *Cyclamen persicum *[41], *Vitis vinifera *[38], and embryogenic cells of *Medicago truncatula *[39]. GST has diverse functions including detoxification of xenobiotics and protection against oxidative stresses, developmental processes and cell cycle [57,64] and may also have a possible role in detoxifying excessive amounts of auxin [65]. Another callus-enriched protein (spot 1752) involved in stress response was ascorbate peroxidase (APX). Similar to our results, differential accumulation of APX has been reported in *Vitis vinifera *embryogenic and non-embryogenic calli [38,66]. By converting H_2_O_2 _to water, APX contributes to scavenging excess H_2_O_2 _during oxidative stresses [67]. Reactive oxygen species (ROS) like H_2_O_2 _cause direct damage to the cellular membranes and oxidation of biological molecules (nucleic acids and proteins) and finally cell death, therefore, plant cells regulate ROS levels through sophisticated mechanisms [68]. Oxidative stress imposed by increased levels of ROS has been reported to improve SE in many plant species [38,69,70]. Spot 2150, which was matched to copper chaperone, significantly accumulated in developed calli. The altered abundance of this protein has also been reported during SE in *Medicago truncatula *[7]. Since free copper is highly reactive and toxic, copper chaperones are involved in intracellular trafficking and loading of copper into copper-containing proteins [71]. Cu/Zn superoxide dismutases are enzyme classes that depend on copper for their catalytic activity. Accumulation of oxidative stress related proteins may be an essential part of stress induced SE and would likely enhance somatic embryo development.

### Protein synthesis and processing

Protein synthesis and processing is necessary for accompanying somatic to embryogenic transition. Synthesis of new proteins and removal of old and unnecessary proteins are a prerequisite for the establishment of a new cell phenotype. The intracellular proteolysis is predominantly mediated by ubiquitin 26S proteasome machinery [72]. By eliminating the abnormal proteins, ubiquitin 26S proteasome system contributes to stress responses. The ubiquitin-proteasome pathway can be regulated at the level of ubiquitination or at the level of proteasome activity [73]. Consistently, we found over accumulation (more than 4 to 5-fold) of a regulatory component of 26S proteasome (spots 1036 and 1042) in NC and NEC. This implies the possible role of proteasome machinery in callus establishment through removal of corm associated proteins and proteins that are no longer needed. Changes in proteasome components has also been reported during somatic and zygotic embryogenesis in other species [39,40,43,69,74]. Spot 1816 matched to cysteine proteinase inhibitor 6. Protease inhibitors control protease activities and could thus regulate protein turnover during SE. Cysteine proteases constitute a large family of proteins that function in programmed cell death (PCD), therefore cysteine proteinase inhibitor may play a role in regulation of PCD during embryonic patterning.

Spot 1199, which was matched to chloroplastic elongation factor Tu, specifically accumulated in nodular calli. This implies the possible role of this protein in early stage of SE, and suggesting more active protein synthesis in chloroplast at this stage of embryo development. Spot 1868, which was identified as translation initiation factor eIF-4 gamma, was highly accumulated in both developed calli compared with corm explant. A little decrease was seen in NC compared with NEC. Several of the differentially accumulated proteins are known as chaperonin [75], including chaperonin containing t-complex polypeptide 1 (spot 641), chaperonin (spots 686 and 727), t-complex protein 1 (680), and Rubisco large subunit-binding protein (RuBP) (676). These proteins help newly synthesized proteins to fold and minimize protein aggregation upon stresses [76,77]. RUBP is a 60 kDa molecular chaperone that specifically involved in Rubisco complex assembly in chloroplast.

### Carbohydrate and energy metabolism

Adaptation to environmental conditions in plant cells is usually accompanied by changing the gene expression and reorganizing metabolic pathways and physiological processes [57]. In this study based on functional classification, proteins involved in metabolic and energy processes comprised the third class of the differentially modulated proteins (17%). Three of the proteins of this class constitute the enzymes involved in glycolysis (spot 621, phosphoglyceromutase), tricarboxylic acid cycle, TCA, (spot 275, aconitase), and pentose phosphate pathway (spot 1756, 6-phosphogluconolactonase). The change in glycolytic and TCA cycle enzymes during zygotic embryogenesis was reported [78], which suggesting more active energy metabolism during embryogenesis. In this study, aconitase increased significantly (up to 8-fold) in NEC and NC compared with their original corm. Lyngved *et al. *[42] also found the accumulation of aconitase during SE in *Cyclamen persicum*. Aconitase regulates carbon flow between TCA cycle and the sucrose synthetic pathway [79] and may also serve as a sensor for oxidants [80]. Fructokinase-2 (spot 1443) exclusively increased in developed calli compared with corm explant. Differential accumulation of fructokinase has already been reported in Valencia sweet orange SE [44] and embryogenic calli treated with 2,4-D [45]. In plants, fructokinases serve as a gateway for fructose metabolism [81] and specifically catalyze phosphorylation of fructose to fructose-6-phosphate. Fructose-6-phosphate is used as a main substrate for several metabolic pathways including starch biosynthesis, glycolysis, and oxidative pentose phosphate. Recently, it was reported that upon GA treatment, fructokinase accumulates in germinating rice seeds, which implies the possible role in dormancy breaking [82].

Spot 1756 was identified as 6-phosphogluconolactonase which catalyzes the hydrolysis of 6-phosphogluconolactone to the sugar acid 6-phosphogluconate as a part of pentose phosphate pathway. It was significantly decreased in both developed calli. dTDP-glucose 4-6-dehydratase (spot 1136) showed a slight decrease in abundance in NC compared with NEC. dTDP-glucose 4-6-dehydratase was first identified in Salmonella [83] and functions in biosynthesis of cell wall polysaccharides. Up regulation of its transcript has been shown in senescent leaves of rice [84]. A deeper analysis of the carbohydrate metabolism related proteins identified in this study suggests that they may play a role in regulating carbon partitioning between different metabolic processes during SE.

One of the differentially accumulated spots (spot 1147) was identified as GDP-D-mannose-3',5'-epimerase (GME). GME appeared to be absent in 2-DE map of the corm explant. It showed a significant increase in embryogenic calli compared with non-embryogenic calli. GME catalyzes the conversion of GDP-D-mannose to GDP-L-galactose, and therefore represents the intersection between L-ascorbate and cell wall polysaccharide biosynthesis [85]. It has been shown that GME is a key regulator of ascorbate biosynthesis pathway and fine-tunes the balance between ascorbate and cell wall monosaccharide biosynthesis [85,86]. Ascorbate is one of the major antioxidants that protects the plant cells against reactive oxygen species (ROS) generated during physiological processes and many biotic and abiotic stresses [85]. Ascorbate serves as a reducing substrate for ascorbate peroxidase (APX), which catalyzes the conversion of H_2_O_2 _to water and generates monodehydroascorbate (MDHA) [87]. Some MDHA radicals spontaneously dismutate to ascorbate and dehydroascorbate (DHA). DHA is reduced to ascorbate in a reaction catalyzed by dehydroascorbate reductase, using glutathione as a specific electron donor [88]. Differential accumulation of GME, as key regulator of ascorbate biosynthesis pathway, and ascorbate peroxidase during early stage of SE imply the possible role of the ascorbate metabolism in scavenging the ROS that might be produced during the process and likely to play an important role in early stage of embryo development [89].

### Secondary metabolism

Among the 50 MS-analyzed proteins in this study, three (Spots 1622, 1644, and 1596) were identified as isoflavone reductase-like (IRL) proteins (1, 5). They appeared reproducibly in developed calli and were not detected in corm explant gels. IRLs have been reported to be increased differentially in embryogenic cell suspension of cowpea [74], embryogenic cells of *Medicago truncatula *[39], and embryogenic calli of *Vitis vinifera *[66]. IRL specifically catalyzes stereospecific reduction of isoflavones in a NADPH-dependent reaction to (3R)-isoflavanones [66]. In previous studies, it was shown that IRL expression is closely correlated with glutathione availability: it is persistently induced in seedlings of maize where glutathione content is about four-fold lower than that of control, and vice versa. This glutathione-dependent regulation indicates that maize IRL may play a crucial role in establishment of a thiol-independent response to oxidative stress under glutathione shortage conditions [90]. Additionally, expression of the IRL gene was demonstrated to be induced by wounding and pathogen infection [91]. It has been reported that IRL accumulates in GA treated germinating rice seeds and is significantly repressed by ABA [82]. In current study, IRLs accumulation in the initial stage of SE indicates their critical role in SE.

One of the most interesting proteins identified in this study was caffeoyl-CoA O-methyltransferase (CCOMT, spot 1750). CCOMT was accumulated in developed calli and was significantly decreased in NC compared to NEC. CCOMT catalyzes the conversion of caffeoyl-CoA to methylated lignin precursors in lignin biosynthesis pathway [92]. Down regulation of CCOMT in alfalfa led to reduced lignin levels and accumulation of soluble caffeic acid β-D-glucoside [93]. The increase in CCOMT abundance in NEC calli may indicate an increase in cell wall lignification and subsequent inhibition of SE. In addition to developmental lignification, lignin biosynthesis in tissue culture systems is stimulated by alteration in growth regulators, water stress, and fungal elicitors [94]. To our knowledge, differential CCOMT accumulation was not reported in the context of SE. Differential regulation of enzymes involved in secondary metabolism suggests a specific role for secondary metabolic pathways during SE. However, further experiments will be required to determine whether any of these proteins are truly involved in SE.

### Nitrogen metabolism

Spots 1297 and 1188 matched to glutamine synthetase (GS) and glutamine synthetase precursor, respectively. They significantly increased in developed calli. It has been shown that glutamine has an important role in proliferation and development of somatic embryos in different species [95-97]. GS catalyzes the amidation reaction of glutamate to glutamine [98]. It seems that during SE a switch takes place in the nitrogen metabolism so that glutamine synthetase/glutamate synthase cycle is the prominent pathway in non-embryogenic cells and germinating embryos whereas during globular and elongated stage embryos ornithine cycle is enhanced and predominant [96]. In an effort Higashi and colleagues [95] studied the expression of the three isoforms of GS (CGS102, CGS103 and CGS201) during somatic and zygotic embryogenesis in carrot. They found that transcript levels of CGS102 and CGS201 accumulate during the early stages of SE and developing seeds, while the CGS103 transcript only expresses in later stages of seed development and senescent leaves and is completely absent in somatic embryos and young leaves. In previous work by Sghaier-Hammami et al. [99], GS was shown to be accumulated in somatic embryos compared to zygotic embryos in date palm. Spot 2192 was identified as Cys/Met metabolism PLP-dependent enzyme family protein. It had significantly lower abundance in NC compered to NEC. This may indicate the possible inhibitory role of this protein on somatic embryogenesis.

There are also candidate proteins in the list of the identified proteins that did not reside in these five functional groups, for example, spot 297, which was identified as cell division control protein 48 homolog A (CDC48). It has been reported that cell cycle genes play a key role in SE [100]. CDC48 is a conserved homohexameric AAA-ATPase chaperone required for a variety of cellular processes. There are several reports demonstrating that CDC48 is critical for cytokinesis, cell expansion, and differentiation in plants [101]. Spot 896 matched to RNA binding protein 45 (RBP45). RBP45 increased significantly in non-embryogenic and embryogenic calli.

## Conclusions

In conclusion, this is the first proteomics analysis that examines the proteomic changes that occur during induction of SE in saffron. 2-DE combined to mass spectrometry led to the identification of several different functional categories of proteins that might be involved in SE. Our results showed that diverse molecular and biochemical processes are affected during SE. The proteome pattern of early explants was significantly different compared to its resultant non-embryogenic (NEC) and embryogenic calli (NC), which points out the necessity for global reprogramming in gene expression and protein complement before gaining the potential for SE. By focusing on specifically accumulated proteins, we aimed to identify proteins which their expression is necessary for somatic to embryogenic transition. The proteome pattern differences between NEC and NC indicate that full reprogramming was not taken place in NEC. There were some polypeptides in different regions of NEC gels that were not detected or had low abundance in NC gels; this may indicate the possible inhibitory effects of these proteins on SE. Of the 36 candidate proteins, sixteen were unique to developed calli. Three proteins were appeared to be increased in NC compared to NEC, and one protein (spot 1199) was increased only in NC. Two proteins (spots 686 and 1756) were reproducibly decreased in developed calli. The physiological and biochemical roles of these differentially modulated proteins are complex, and may sometimes conflict with each other. The analysis of the differentially modulated proteins in the developed calli suggests that the embryogenic status is related to a better capability of regulating oxidative stresses, both by fine-tuning of the ROS-scavenging system (mainly through ascorbate-glutathione cycle) and the maintaining protein structure by means of HSPs.

## Abbreviations

2-DE: two-dimensional gel electrophoresis; SE: somatic embryogenesis; 2,4-D: 2,4-dichlorophenoxy acetic acid; Kin; kinetin; NAA: naphthalene acetic acid; IAA: indole acetic acid; ABA: abscisic acid; ACN: acetonitrile; TCA: trichloroacetic acid; SDS: sodium dodecyl sulfate; CBB: Coomassie brilliant blue DMSO: dimethyl sulfoxide; TFA: trifluoroacetic acid; MALDI-TOF/TOF: matrix-assisted laser desorption ionization-time of flight/time of flight; MS: mass Spectrometry; MS/MS: tandem mass spectrometry; PMF: peptide mass finger printing; PTM: posttranslational modification; GST: glutathione S-transferase; APX: Ascorbate peroxidase; GS: glutamine synthetase; MDHA: monodehydroascorbate; DHA: dehydroascorbate.

## Competing interests

The authors declare that they have no competing interests.

## Authors' contributions

GS conceived and designed the experiment and carried out tissue culture. EV involved in sample preparation. GS and JG performed 2-DE and wrote the manuscript. HE participated in the conceiving, design, and coordination of this study. BG helped the research. All authors read and approved the final manuscript.
